# Just-Released JCR Impact Factor Shows Strong and Steady Increase for ABC Cardiol - 1.679 - A New Historical Record

**DOI:** 10.5935/abc.20190135

**Published:** 2019-07

**Authors:** Carlos E. Rochitte

**Affiliations:** Instituto do Coração - Incor, São Paulo, SP - Brazil

**Keywords:** Bibliometrics, Journal Impact Factor, Periosicals as Topic, Scimago

Dear friends from the Cardiology and Cardiovascular scientific community in Brazil and abroad, please allow me this short editorial with extraordinary news on the ABC Cardiol Journal, accompanied by data, of course.

We are delighted to announce that our peer-reviewed journal of The Brazilian Society of Cardiology, the ABC Cardiol (Arquivos Brasileiros de Cardiologia), has seen significant increase in its impact factor rating in the latest Journal of Citation Reports (JCR) release. Our impact factor went up from 1.318, released last year (2017 rate) to 1.679 this year (2018 rate), a 27% increase in one year and the highest impact factor ever for ABC Cardiol.

**Figure 1:**
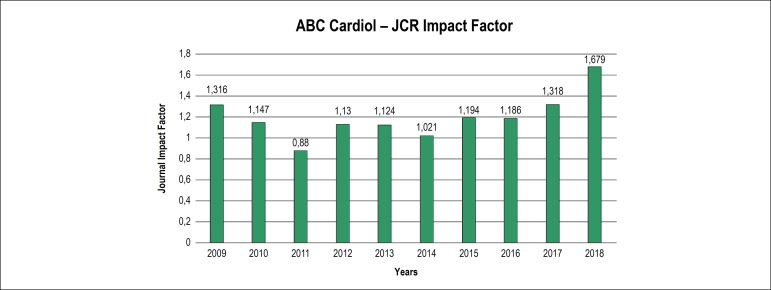
ABC Cardiol JCR Impact Factor from 2009 to 2018.

Source: JCR^1^

**Figure 2:**
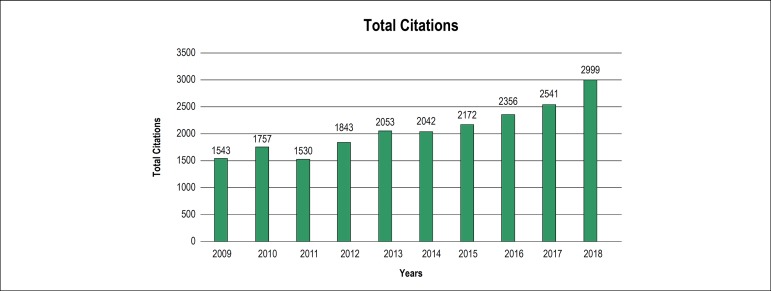
ABC Cardiol Total Citations per Year

Source: JCR^1^

We can see a clear and steep increase of the impact factor in Figure 1,^1^ breaking the barrier of 1,5 impact factor. Articles published in the ABC Cardiol received a staggering 3000 citations in 2018 alone (Figure 2).^1^ Another barrier that was broken by ABC Cardiol this year is the third Quartile. We are now ranked at the Q3 of all 136 Cardiac and Cardiovascular System journals in the World (Table 1).^1^ ABC Cardiol galvanized his position of the highest ranked Journal in Cardiology and Cardiovascular Science in Latin America, position that was reached and maintained since 2014 (see Table 2).^2^

**Table 1:** ABC Cardiol Quartiles Rank from 2009 to 2018. Source JCR^1^

JCR year	Cardiac and Cardiovascualr systems		
	Rank	Quartile	JIF Percentile
2018	98/136	Q3	23.309
2017	106/128	Q4	17.578
2016	107/126	Q4	15.476
2015	97/124	Q4	22.177
2014	100/123	Q4	19.106
2013	96/125	Q4	23.600
2012	91/124	Q3	27.016
2011	98/117	Q4	16.667
2010	87/114	Q4	24.123
2009	63/95	Q3	34.211

**Table 2:** Rank in Cardiology and Cardiovascular Science Journals in Latin America. Source SCIMAGO^2^

Rank	Title	SJR	SJR Quartile	H index	Total Docs. (2018)	Total Docs. (3years)	Total Refs.	Total Cites (3years)	Citable Docs. (3years)	Cites / Doc. (2years)	Ref./Doc.	Country
1	Arquivos Brasileiros de Cardiologia	0,407	Q3	45	250	595	5472	592	418	1,41	21,89	Brazil
2	Brazilian Journal of Cardiovascular Surgery	0,324	Q3	22	109	290	2058	234	237	0,83	18,88	Brazil
3	Jornal Vascular Brasileiro	0,158	Q4	13	61	178	1146	71	162	0,42	18,79	Brazil
4	Archivos de Cardiologia de Mexico	0,142	Q4	16	94	195	2043	46	174	0,26	21,73	Mexico
5	Revista Argentina de Cardiologia	0,127	Q4	9	109	368	1375	18	159	0,1	12,61	Argentina
6	Revista Latinoamericana de Hipertension	0,124	Q4	5	95	53	3091	25	53	0,55	32,54	Venezuela
7	Revista Colombiana de Cardiologia	0,117	Q4	8	135	411	3196	54	386	0,12	23,67	Colombia
8	Revista Brasileira de Cardiologia Invasiva	0,113	Q4	7	0	100	0	5	81	0	0	Brazil
9	Revista de la Federacion Argentina de Cardiologia	0,112	Q4	4	30	158	449	9	124	0,04	14,97	Argentina
10	Revista Mexicana de Angiologia	0,112	Q4	3	23	74	288	3	62	0,05	12,52	Mexico
11	Insuficiencia Cardiaca	0,109	Q4	5	21	77	592	3	67	0,02	28,19	Argentina
12	Revista Mexicana de Cardiologia	0,104	Q4	4	22	94	638	10	86	0,09	29	Mexico
13	Revista Mexicana de Enfermeria Cardiologica	0,101	Q4	2	0	29	0	0	24	0	0	Mexico

One important aspect of the progression of our JCR impact factor is depicted in the Table 3.^1^ While in the same period of 2017 to 2018, most of Cardiology and Cardiovascular Science and Medical Journals in Brazil only maintained or even decreased their impact factor, ABC Cardiol steadily increased in a significantly amount its impact factor, only followed by a basic research journal. So, in our scientific community in Latin America ABC Cardiol has established itself as the main reference journal for Cardiology and Cardiovascular Science.

**Table 3:** JCR impact factor for 2017 and 2018 for Brazilian Journals (Cardiovascular Field, Medicine and Basic Research). Source JCR^1^

Journal	Impact Factor 2017	Impact Factor 2018
Arquivos Brasileiros de Cardiologia	1,318	1,679
Brazilian Journal of Cardiovascular Surgery	0,805	0,796
Brazilian Journal of Medical and Biological Research	1,492	1,850
Clinics	1,245	1,127
Memorias do Instituto Oswaldo Cruz	2,833	2,368
Revista da Associacao Medica Brasileira	0,736	0,801
Sao Paulo Medical Journal	1,063	1,088

The rise in impact factor means that our research community is recognizing the articles being accepted and published in ABC Cardiol journal as relevant and impactful science. This accomplishment fulfills ABC Cardiol mission that is to promote new knowledge and publish the latest research, emerging technologies and breakthrough advances in Cardiology and cardiac diseases.

ABC Cardiol journal is free and open access journal that can be viewed, downloaded and accessed by mobile app from anywhere in the World. The readers can interact with editorial board members and authors through social media (Facebook, Tweeter, Instagram, etc) and through movie posts in our newly re-designed web-portal.

This important step on the ABC Cardiol progression toward a higher scientific rank was only possible by a continuous editorial policy that started several years back with Dr. Luis Felipe Moreira, former Editor-in-Chief, to whom we are deeply grateful. Our international and talented associate editors’ team, dedicated editorial board and reviewers are by far the main reason that led ABC Cardiol to his current position. The strong support from our Brazilian Society of Cardiology board of directors to the ABC Cardiol has been crucial to achieve our goals of increasing our impact and presence in the scientific community. Our dedicated team of editorial assistants that does not measure the efforts to achieve timely and high quality publication is of paramount importance to our ABC Cardiol journal operation. For me as editor-in-chief, running ABC Cardiol and working together with this great team of professionals in our office in São Paulo and in Rio de Janeiro has been a blessing. Thank you so much for this delightful experience.

Our editorial policy is focused in the scientific quality of submitted manuscripts and the novelty they bring to the field. We welcome new and revolutionary ideas brought to light by rigorous scientific method in well-written manuscripts. Our main language is English, but we accept and publish all articles bilingually, English and Portuguese.

We have editorial comments for all published original articles, which place the new science in context with the specific research field. ABC Cardiol is also the home of all clinical guidelines, statements and expert consensus endorsed by the Brazilian Society of Cardiology. All guidelines are now published in Portuguese and English and within the body of the Journal. With these recent improvements in our editorial policy and continuous support from our scientific community and post-graduation programs in Brazil we hope we can break another impact factor barrier next year. Thank you all very much.
